# Preparation of spirocyclic oxindoles by cyclisation of an oxime to a nitrone and dipolar cycloaddition

**DOI:** 10.3762/bjoc.21.146

**Published:** 2025-09-11

**Authors:** Beth L Ritchie, Alexandra Longcake, Iain Coldham

**Affiliations:** 1 Chemistry, School of Mathematical and Physical Sciences, University of Sheffield, Sheffield S3 7HF, UK; 2 Chemistry, School of Natural and Environmental Sciences, University of Newcastle, Newcastle NE1 7RU, UK

**Keywords:** cascade, cycloaddition, oxindole, spirocycle, stereochemistry

## Abstract

Oxindoles are an important class of compounds with significant biological activities. Spirocyclic derivatives are present in a variety of natural products. We describe here the formation of spirooxindoles using an intermolecular nitrone cycloaddition reaction. The nitrone dipole was prepared in situ by cyclisation of an oxime, itself prepared in situ from an aldehyde. The stereochemistry of one of the spirooxindoles was determined by single crystal X-ray diffraction studies via crystallisation using encapsulated nanodroplet crystallisation (ENaCt) protocols. The chemistry involves cascade or tandem condensation, cyclisation, and cycloaddition as an efficient strategy for the rapid formation of complex spirocyclic products that could have value for the formation of novel, bioactive oxindoles.

## Introduction

**Figure 1:**
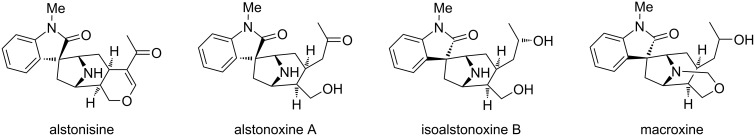
Representative oxindole alkaloids.

The *Alstonia* alkaloids are derived from evergreen trees found in tropical and subtropical regions of Africa, Australia, Central America, Polynesia, and Southeast Asia. Plants of the genus *Alstonia* have been used in traditional medicines to treat a wide variety of conditions, including coughs, dysentery, fever, malaria, rheumatism, sore throats, toothache, ulcers, and snake bites [1]. The medicinal qualities of these plants are ascribed to the presence of alkaloids, and they are rich in many different indole and oxindole alkaloids [2]. About 100 *Alstonia* alkaloids are known, with more being isolated on a regular basis [3–4]. Representative examples are shown in Figure 1. The alkaloid alstonisine has antimalarial activity, with IC_50_ 7.6 μM against *Plasmodium falciparum* [5]. However, the biological activities of most of the *Alstonia* alkaloids are currently unknown, despite the potential of spirooxindoles for drug discovery [6]. This is in part due to limitations in the quantities obtained in the extraction processes. Other selected examples shown in Figure 1 all contain the same spirocyclic oxindole with a bridged azabicyclo[3.2.1]octane moiety.

Several methods to access spirocyclic oxindole alkaloids have been reported [7–13]. Of note, Cook and co-workers described an elegant approach using tryptophan as a starting material and an oxidative rearrangement of an indole that allows access to a variety of spirooxindole alkaloids from the *Alstonia* genus [14].

**Scheme 1:**
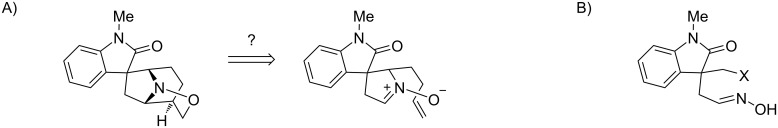
Proposed synthetic approach.

An efficient way to construct cyclic nitrogen-containing compounds makes use of intramolecular 1,3-dipolar cycloaddition reactions [15], including examples with nitrone ylides [16–22]. Our research group has exploited this approach for the synthesis of alkaloids such as myrioxazine A and aspidospermidine [23–24]. With a nitrone 1,3-dipole, access to 1,3-amino-alcohol functionality is possible. This arrangement is present in many *Alstonia* alkaloids, and we envisaged using this cycloaddition chemistry to set up the bridged amine ring system found in these natural products. Bridged and spirocyclic ring systems are known to be accessible by this chemistry [25–26], as demonstrated in the formation of the core of the *Daphniphyllum* alkaloids [27]. The *Alstonia* alkaloids shown in Figure 1 consist of a bridging nitrogen atom in an azabicyclo[3.2.1]octane that is spiro-fused with an oxindole. A retrosynthetic analysis suggested the possibility to access the core of this ring system with the nitrone intermediate shown in Scheme 1A. As a model study, we describe here efforts towards an intermolecular variant with the oxindole shown in Scheme 1B; in this case a leaving group X will be displaced by the oxime to give an intermediate nitrone that can be explored for its ability to undergo dipolar cycloaddition chemistry.

## Results and Discussion

**Scheme 2:**
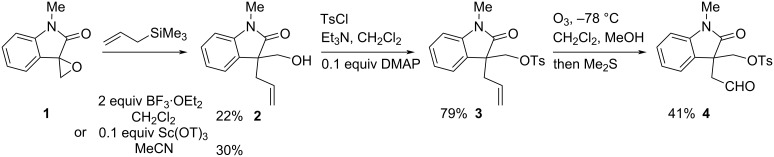
Preparation of the aldehyde **4**.

The oxindole core was prepared from isatin following known chemistry to give the spirocyclic epoxide **1** (Scheme 2) [28]. This was subjected to regioselective ring-opening with allyltrimethylsilane in the presence of a Lewis acid. The use of BF_3_·OEt_2_ gave a low yield of the desired alcohol **2** [29]. This was improved slightly with Sc(OTf)_3_ as the Lewis acid, which could be used substoichiometrically [30]. The alcohol **2** was converted to the tosylate **3** and subsequent ozonolysis gave the aldehyde **4**.

The aldehyde **4** could now be tested in the cascade chemistry. This entails the addition of hydroxylamine to form the oxime, followed by cyclisation (with displacement of the tosylate) to give the nitrone for the desired dipolar cycloaddition reaction. Related chemistry (without the oxindole) with a halide leaving group and activated, electron-poor dipolarophiles has been reported [31–32].

**Scheme 3:**
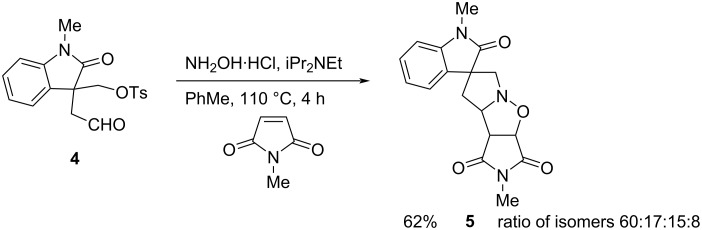
Cycloaddition with *N*-methylmaleimide.

**Figure 2:**
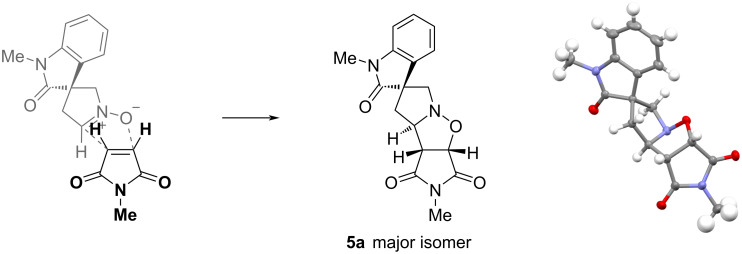
Orientation for the cycloaddition (left) and the crystal structure of the major stereoisomer **5a** (right) with the anisotropic displacement parameters drawn at 50% (oxygen – red; nitrogen – blue; carbon – grey; hydrogen – pale green).

Treatment of aldehyde **4** with hydroxylamine in toluene at 60 °C for 1–3 h resulted in loss of the aldehyde as judged by ^1^H NMR spectroscopy of the crude mixture. When this was carried out in the presence of *N*-methylmaleimide with the mixture being heated under reflux for 3–4 h, we were pleased to obtain the spirooxindole product **5**, which was formed as a mixture of stereoisomers (Scheme 3). The ratio of isomers was determined by ^1^H NMR spectroscopy and the major isomer could be isolated after careful column chromatography on silica gel. The structure of this isomer could not be ascertained with certainty from NOESY analysis and initial crystallisation attempts were unsuccessful. However, through use of encapsulated nanodroplet crystallisation (ENaCt), a high-throughput crystallisation technique which controls the rate of evaporation from a nanolitre solution of analyte encased within oil [33], suitable single crystals were obtained. The X-ray analysis allowed the determination of the relative stereochemistry, as shown in Figure 2. Compound **5a** crystallised as a racemic mixture in the space group *P*21/*c*. The major stereoisomer has the ring-junction NC–H bond, the imide carbonyls, and the oxindole carbonyl all *cis* to one another across the two newly formed rings. This must arise from a preference for an *exo* transition state that places the incoming maleimide away from the oxindole carbonyl.

**Scheme 4:**
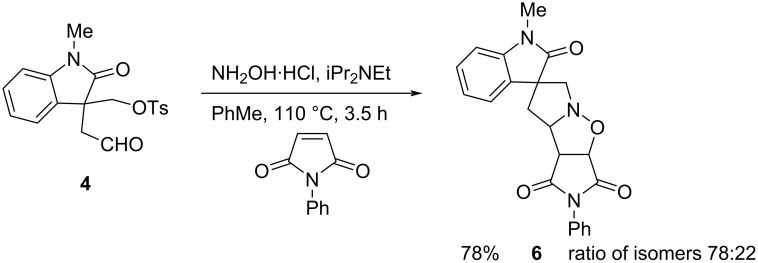
Cycloaddition with *N*-phenylmaleimide.

The cascade chemistry allows the formation of two new rings and a complex polycyclic product from relatively simple starting materials. To test the scope of the chemistry, we screened several other dipolarophiles for this transformation. As expected, the related *N*-phenylmaleimide resulted in the formation of the analogous *N*-phenyl cycloadduct **6** (Scheme 4). The yield of cycloadduct **6** was good and there were only two stereoisomers obtained that were inseparable. Based on the result with the *N*-methyl analogue, we predict that the major isomer has the same stereochemistry as that of compound **5a**.

**Scheme 5:**
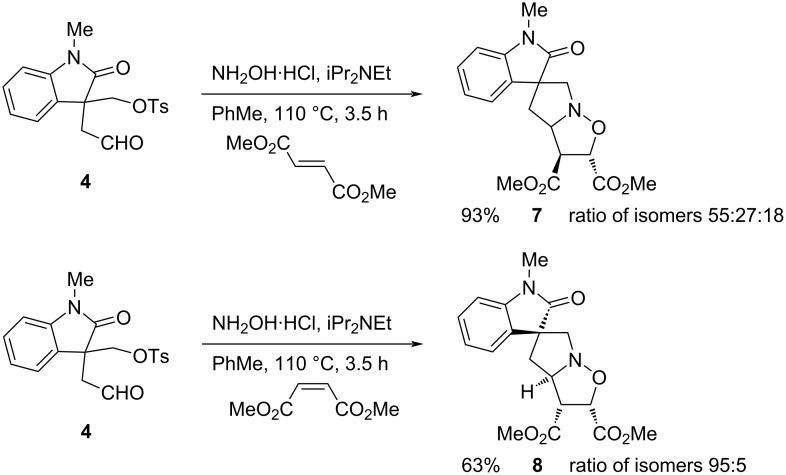
Cycloaddition with dimethyl fumarate and dimethyl maleate.

The dipolarophile dimethyl fumarate was also successful and gave a mixture of the stereoisomeric cycloadducts **7** (Scheme 5). The yield was high and three inseparable stereoisomers could be detected by ^1^H NMR spectroscopy. In each case we assume that the methyl ester groups are *trans* to one another due to the expected concerted nature of the dipolar cycloaddition process. In contrast, the dipolarophile dimethyl maleate gave predominantly one stereoisomer of the cycloadduct **8** (ratio of isomers 95:5) (Scheme 5). The stereochemistry of the major isomer was assigned from NOESY analysis, that indicated the methyl ester groups were *cis* to one another and *cis* to the ring junction NC–H. The relative stereochemistry of the oxindole quaternary stereocentre was less obvious from this method, but the coupling constants of the aliphatic ring protons in the ^1^H NMR spectrum had similar values to those in the adduct **5a**, suggesting that the same stereochemical outcome predominates. There are two possible *exo* transition states, both of which avoid steric (and electronic clash) of the dipolarophile carbonyls with the oxindole. Of these, the preference appears to be for the dipolarophile carbonyl groups, in this case for dimethyl maleate, to approach the nitrone on the side opposite the oxindole carbonyl (in a similar way to that shown in Figure 2 for the maleimide).

## Conclusion

In conclusion, we have demonstrated the feasibility of our approach to the core of several *Alstonia* alkaloids by using a cascade cyclisation–dipolar cycloaddition approach. This generates the desired spirooxindole connected to a bicyclic amine, with functionality related to the natural products. It is likely that unactivated dipolarophiles could be successful in related intramolecular cycloadditions [18]. Hence this approach could allow access the spirooxindole and bridged azabicyclic ring system found in alstonoxine A and related alkaloids.

## Experimental

Chemicals described were obtained from commercial suppliers and were used without further purification. Solvents were obtained from a Grubbs dry solvent system. Thin-layer chromatography was performed on Merck silica gel 60F254 plates and visualised by UV irradiation at 254 nm or by staining with an alkaline KMnO_4_ dip. Flash column chromatography was performed using silica gel (40–63 micron mesh). Infrared spectra were recorded on a Perkin Elmer Spectrum RX Fourier Transform–IR System and only selected peaks are reported. ^1^H NMR spectra were recorded on a Bruker AC400 (400 MHz) instrument. Chemical shifts are reported in ppm with respect to the residual solvent peaks, with multiplicities given as s = singlet, d = doublet, t = triplet, q = quartet, m = multiplet, br = broad. Coupling constants (*J* values) are quoted to the nearest 0.5 Hz with values in hertz (Hz). ^13^C NMR spectra were recorded on the above instrument at 100 MHz. Low- and high-resolution (accurate mass) mass spectra were recorded on a Walters LCT instrument using electrospray ionisation (ESI).

### Synthesis of the aldehyde **4**

Sc(OTf)_3_ (0.63 g, 1.3 mmol) was added to epoxide **1** [28] (2.2 g, 12.7 mmol) and allyltrimethylsilane (6.1 mL, 38 mmol) in dry MeCN (90 mL) at 0 °C. After 1 h, saturated aqueous NaHCO_3_ (50 mL) was added, and the mixture was extracted with EtOAc (2 × 50 mL). The combined organic layers were washed with brine (20 mL), dried (MgSO_4_), filtered and evaporated. The residue was purified by column chromatography on silica gel, eluting with EtOAc/petrol 1:2, to give the alcohol **2** (824 mg, 30%) as an amorphous solid; data as reported [30].

Et_3_N (1.5 mL, 10.8 mmol) was added to the alcohol **2** (782 mg, 3.6 mmol), TsCl (1.03 g, 5.4 mmol) and DMAP (132 mg, 1.1 mmol) in CH_2_Cl_2_ (45 mL) at room temperature. After 4 d, saturated aqueous NaHCO_3_ (45 mL) was added and the layers were separated. The organic layer was dried (MgSO_4_), filtered and evaporated. The residue was purified by column chromatography on silica gel, eluting with EtOAc/petrol 1:4, to give the tosylate **3** (1.30 g, 79%) as an amorphous solid; *R*_f_ 0.57 (EtOAc/petrol 1:1); IR (film, cm^−1^) ν_max_: 2943, 1708, 1647, 1611, 1599, 1494, 1468, 1455, 1425, 1359, 1314, 1292, 1258, 1178, 1119, 1023, 976, 936, 885, 831, 816, 776, 752; ^1^H NMR (400 MHz, CDCl_3_) δ 7.66–7.59 (m, 2H), 7.33–7.27 (m, 3H), 7.19 (dd, *J* = 7.5, 1.0 Hz, 1H), 7.04 (td, *J* = 7.5, 1.0 Hz, 1H), 6.81 (d, *J* = 7.5 Hz, 1H), 5.38–5.25 (m, 1H), 4.97 (dq, *J* = 17.0, 1.5 Hz, 1H), 4.90 (dd, *J* = 10.0, 1.5 Hz, 1H), 4.27 (d, *J* = 9.5 Hz, 1H), 4.13 (d, *J* = 9.5 Hz, 1H), 3.16 (s, 3H), 2.61–2.45 (m, 2H), 2.44 (s, 3H); HRESIMS (*m/z*): [M + H]^+^ calcd for C_20_H_22_NO_4_S, 372.1270; found: 372.1281; ^1^H NMR data as reported [30].

Ozone was bubbled through a solution of **3** (1.30 g, 3.74 mmol) in CH_2_Cl_2_ (22 mL) and MeOH (22 mL) at −78 °C. After 1 h, excess ozone was removed by bubbling argon gas through the solution. After 5 min, dimethyl sulphide (51.3 mL, 693 mmol) was added, and the mixture was warmed to room temperature and stirred. After 16 h, the solvent was evaporated and the residue was purified by column chromatography on silica gel, eluting with EtOAc/petrol 1:1. The product was recrystallised from *n*-hexane/CH_2_Cl_2_ to give aldehyde **4** (562 mg, 41%) as needles; mp 94–96 °C; *R*_f_ 0.35 (EtOAc/petrol 1:1); IR (film, cm^−1^) ν_max_: 2942, 1712, 1614, 1581, 1495, 1472, 1260, 1190, 1177, 1122, 1096, 1064, 1019, 815, 785, 755; ^1^H NMR (400 MHz, CDCl_3_) δ 9.44 (t, *J* = 1.0 Hz, 1H), 7.69–7.62 (m, 2H), 7.36–7.28 (m, 3H), 7.20 (dd, *J* = 7.5, 1.0 Hz, 1H), 7.02 (td, *J* = 7.5, 1.0 Hz, 1H), 6.86 (d, *J* = 7.5 Hz, 1H), 4.27 (d, *J* = 9.5 Hz, 1H), 3.97 (d, *J* = 9.5 Hz, 1H), 3.23 (s, 3H), 3.17 (dd, *J* = 18.0, 1.0 Hz, 1H), 2.99 (dd, *J* = 18.0. 1.0 Hz, 1H), 2.44 (s, 3H); ^13^C NMR (101 MHz, CDCl_3_) δ 196.7, 174.9, 145.0, 143.6, 132.0, 129.8, 129.0, 127.8, 127.6, 124.0, 123.7, 108.4, 72.0, 48.4, 46.2, 26.4, 21.5; NMR data as reported (no lit. mp) [30].

### Cycloadditions with the aldehyde **4**

Aldehyde **4** (500 mg, 1.44 mmol), hydroxylamine hydrochloride (160 mg, 2.30 mmol), and iPr_2_NEt (0.48 mL, 2.76 mmol) in PhMe (10 mL) were heated to 60 °C. After 2 h, *N*-methylmaleimide (255 mg, 2.30 mmol) was added and the mixture was heated under reflux. After 3 h, further *N*-methylmaleimide (128 mg, 1.84 mmol) was added, and the mixture was heated under reflux for a further 1 h. The mixture was cooled to room temperature and the solvent was evaporated. The crude product [mixture of isomers (60:17:15:8) – see below] was purified by column chromatography on silica gel, eluting with EtOAc/petrol 1:2.5, to give the oxindole **5** (293 mg, 62%) as an amorphous solid. A portion of the major isomer was isolated as an amorphous solid; data for isomer **5a**: *R*_f_ 0.62 (CH_2_Cl_2_/MeOH 9:1); IR (film, cm^−1^) ν_max_: 2941, 1784, 1694, 1612, 1495, 1471, 1434, 1377, 1352, 1281, 1247, 1181, 1138, 1092, 1072, 1041, 993, 932, 870; ^1^H NMR (400 MHz, CDCl_3_) δ 7.30 (td, *J* = 7.5, 1.0 Hz, 1H), 7.21 (br d, *J* = 7.5 Hz, 1H), 7.07 (td, *J* = 7.5, 1.0 Hz, 1H), 6.84 (br d, *J* = 7.5 Hz, 1H), 5.07 (d, *J* = 7.5 Hz, 1H), 4.25 (ddd, *J* = 11.0, 6.5, 1.5 Hz, 1H), 3.86 (d, *J* = 15.0 Hz, 1H) 3.70 (dd, *J* = 7.5, 1.5 Hz, 1H), 3.42 (d, *J* = 15.0 Hz, 1H), 3.22 (s, 3H), 3.08 (s, 3H), 2.55 (dd, *J* = 13.0, 11.0 Hz, 1H), 2.24 (dd, *J* = 13.0, 6.5 Hz, 1H); ^13^C NMR (101 MHz, CDCl_3_) δ 178.4, 175.2, 174.8, 142.9, 134.2, 128.8, 123.4, 122.3, 108.5, 76.2, 71.9, 65.3, 56.1, 52.9, 42.8, 26.8, 25.3; HRESIMS (*m/z*): [M + H]^+^ calcd for C_17_H_18_N_3_O_4_, 328.1297; found: 328.1292.

The diastereoisomeric ratio was determined from the crude ^1^H NMR spectrum of the crude material by integrating the peaks for OC–H at 5.07 (d, *J* = 7.5 Hz, 0.60H), 5.00 (d, *J* = 8.0 Hz, 0.17H), 5.04 (d, *J* = 8.0 Hz, 0.08H), 5.03 (d, *J* = 7.5 Hz, 0.15H).

Aldehyde **4** (192 mg, 0.55 mmol), hydroxylamine hydrochloride (96 mg, 1.4 mmol), and iPr_2_NEt (0.23 mL, 1.32 mmol) in PhMe (3 mL) were heated to 60 °C. After 30 min, *N*-phenylmaleimide (287 mg, 1.66 mmol) was added, and the mixture was heated under reflux. After 3.5 h, the mixture was cooled to room temperature and the solvent was evaporated. The residue was purified by column chromatography on silica gel, eluting with EtOAc/hexane 1:4 to 1:0, to give the oxindole **6** (140 mg, 78%) as an inseparable mixture of isomers (88:12) as an amorphous solid; *R*_f_ 0.16 (EtOAc/petrol 1:1); IR (film, cm^−1^) ν_max_: 2960, 2874, 1782, 1711, 1697, 1610, 1493, 1468, 1376, 1350, 1266, 1247, 1187, 1086, 1070, 1019, 971, 869, 759; ^1^H NMR (400 MHz, CDCl_3_) δ 7.47–7.30 (m, 6H), 7.25–7.21 (m, 1H), 7.07 (td, *J* = 7.5, 1.0 Hz, 1H), 6.84 (d, *J* = 7.5 Hz, 1H), 5.20 (d, *J* = 7.5 Hz, 0.88H), 5.13 (d, *J* = 7.5 Hz, 0.12H), 4.53 (d, *J* = 7.5 Hz, 0.12H), 4.34 (dd, *J* = 10.5, 6.5 Hz, 0.88H), 3.88 (d, *J* = 15.0 Hz, 1H), 3.83 (d, *J* = 7.5, 1H), 3.45 (d, *J* = 15.0 Hz, 1H), 3.21 (s, 2.68H), 3.19 (s, 0.36H), 2.57 (dd, *J* = 13.0, 10.5 Hz, 1H), 2.25 (dd, *J* = 13.0, 6.5 Hz, 1H); ^13^C NMR (101 MHz, CDCl_3_) δ 178.5, 174.5, 173.9, 142.7, 134.1, 131.4, 129.3, 129.0, 128.7, 126.5, 123.4, 122.3, 108.8, 76.1 (major), 72.2 (major), 70.2 (minor), 65.3, 62.6 (minor), 56.0, 52.9, 42.7, 26.8; HRESIMS (*m/z*): [M + H]^+^ calcd for C_22_H_20_N_3_O_4_, 390.1454; found: 390.1448.

Aldehyde **4** (220 mg, 0.63 mmol), hydroxylamine hydrochloride (88 mg, 1.3 mmol), and iPr_2_NEt (0.26 mL, 1.5 mmol) in PhMe (5 mL) were heated to 60 °C. After 1 h, dimethyl fumarate (273 mg, 1.9 mmol) was added, and the mixture was heated under reflux. After 3.5 h, the mixture was cooled to room temperature and the solvent was evaporated. The crude product [mixture of isomers (55:27:18) – see below] was purified by column chromatography on silica gel, eluting with EtOAc/hexane 1:2, to give the oxindole **7** (213 mg, 93%) as an amorphous solid. A partially separated sample [mixture of isomers (83:17)] was isolated as an amorphous solid and the data are given here: *R*_f_ 0.45 (CH_2_Cl_2_/MeOH 9:1); IR (film, cm^−1^) ν_max_: 2956, 1737, 1707, 1612, 1493, 1474, 1437, 1376, 1352, 1260, 1205, 1129, 1085, 1020, 989; ^1^H NMR (400 MHz, CDCl_3_) δ 7.45 (dd, *J* = 7.5, 1.5 Hz, 1H), 7.30–7.22 (m, 1H) 7.12–7.06 (m, 1H), 6.80 (d, *J* = 7.5 Hz, 1H), 5.22 (d, *J* = 5.0 Hz, 0.83 H), 5.09 (d, *J* = 6.0 Hz, 0.17H), 4.40 (dt, *J* = 10.5, 7.0 Hz, 1H), 4.23 (dd, *J* = 7.0, 5.0 Hz, 0.83H) 4.17–4.08 (m, 0.17H), 3.85 (s, 3H), 3.84 (d, *J* = 18.5 Hz, 1H), 3.71 (s, 3H), 3.70 (d, *J* = 18.5 Hz, 1H), 3.19 (s, 3H), 2.56 (dd, *J* = 13.0, 7.0 Hz, 0.17H), 2.33 (dd, *J* = 13.0, 7.0 Hz, 0.83H), 2.25 (dd, *J* = 13.0, 10.5 Hz, 0.17H), 2.09 (dd, *J* = 13.0, 10.5 Hz, 0.83H); ^13^C NMR (101 MHz, CDCl_3_) δ 180.4, 171.9, 170.4, 143.3, 134.0, 128.2, 124.5, 123.5, 108.0, 77.9 (minor), 76.0 (major), 72.2 (minor), 70.5 (major), 66.0, 56.4, 54.8, 53.1 (major), 53.0 (minor), 52.9 (minor), 52.7 (major), 41.6, 26.5; HRESIMS (*m/z*): [M + H]^+^ calcd for: C_18_H_21_N_2_O_6_, 361.1400; found: 361.1394.

The diastereoisomeric ratio was determined from the ^1^H NMR spectrum of the crude material by integrating the peaks for OC–H at 5.22 (d, *J* = 5.0 Hz, 0.55H), 5.09 (d, *J* = 6.0 Hz, 0.18H), 5.06 (d, *J* = 8.0 Hz, 0.27H).

Aldehyde **4** (500 mg, 1.44 mmol), hydroxylamine hydrochloride (200 mg, 2.9 mmol), and iPr_2_NEt (0.6 mL, 3.5 mmol) in PhMe (7 mL) were heated to 60 °C. After 1 h, dimethyl maleate (0.54 mL, 1.9 mmol) was added, and the mixture was heated under reflux. After 3.5 h, the mixture was cooled to room temperature and the solvent was evaporated. The crude product was purified by column chromatography on silica gel, eluting with EtOAc/hexane 1:2, to give the oxindole **8** (353 mg, 63%) as a 95:5 mixture of isomers an amorphous gum; *R*_f_ 0.28 (EtOAc/petrol 1:1); IR (flm, cm^−1^) ν_max_: 2953, 1738, 1707, 1612, 1493, 1470 1437, 1376, 1352, 1212, 1019; ^1^H NMR (400 MHz, CDCl_3_) δ 7.43 (br d, *J* = 7.5 Hz, 1H), 7.29 (td, *J* = 7.5, 1.0 Hz, 1H), 7.10 (td, *J* = 7.5, 1.0 Hz, 1H), 6.83 (br d, *J* = 7.5 Hz, 1H), 5.14 (d, *J* = 8.0 Hz, 0.95H), 5.09 (d, *J* = 6.0 Hz, 0.05H), 4.48 (td, *J* = 8.0, 5.0 Hz, 0.95H), 4.25 (t, *J* = 6.5 Hz, 0.05H), 4.05 (t, *J* = 8.0 Hz, 0.95H), 3.91–3.65 (m, 2H), 3.79 (s, 3H), 3.74 (s, 3H), 3.22 (s, 3H), 2.45 (dd, *J* = 13.5, 5.0 Hz, 1H), 2.32 (dd, *J* = 13.5, 8.0 Hz, 1H); ^13^C NMR (101 MHz, CDCl_3_, peaks for major isomer) δ 178.1, 170.0, 169.7, 142.7, 133.9, 128.6, 123.6, 123.5, 108.3, 78.7, 67.9, 64.6, 55.4, 53.7, 52.8, 52.7, 39.4, 26.8; HRESIMS (*m*/*z*): [M + H]^+^ calcd for C_18_H_21_N_2_O_6_, 361.1400, found: 361.1404.

## Supporting Information

File 1 ENaCt protocols, X-ray diffraction data for **5a**, and NMR spectra for novel compounds.

## Data Availability

All data that supports the findings of this study is available in the published article and/or the supporting information of this article.

## References

[1] Pan L, Terrazas C, Muñoz Acuña U, Ninh T N, Chai H, Carcache De Blanco E J, Soejarto D D, Satoskar A R, Kinghorn A D (2014). Phytochem Lett.

[2] Tan S-J, Lim J-L, Low Y-Y, Sim K-S, Lim S-H, Kam T-S (2014). J Nat Prod.

[3] Atta-ur-Rahman, Nighat F, Nelofer A, Zaman K, Choudhary M I, DeSilva K T D (1991). Tetrahedron.

[4] Li Z-W, Fan C-L, Sun B, Huang L, Wang Z-Q, Huang X-J, Zhang S-Q, Ye W-C, Wu Z-L, Zhang X-Q (2024). Chem – Eur J.

[5] Cheenpracha S, Ritthiwigrom T, Laphookhieo S (2013). J Nat Prod.

[6] Zhou L-M, Qu R-Y, Yang G-F (2020). Expert Opin Drug Discovery.

[7] Marti C, Carreira E M (2003). Eur J Org Chem.

[8] Sansinenea E, Martínez E F, Ortiz A (2020). Eur J Org Chem.

[9] Chen P, Yang H, Zhang H, Chen W, Zhang Z, Zhang J, Li H, Wang X, Xie X, She X (2020). Org Lett.

[10] Boddy A J, Bull J A (2021). Org Chem Front.

[11] Saleh S K A, Hazra A, Singh M S, Hajra S (2022). J Org Chem.

[12] Asif M, Azaz T, Tiwari B, Nasibullah M (2023). Tetrahedron.

[13] Nam Y, Tam A T, Miller E R, Scheidt K A (2024). J Am Chem Soc.

[14] Stephen M R, Rahman M T, Tiruveedhula V V N P B, Fonseca G O, Deschamps J R, Cook J M (2017). Chem – Eur J.

[15] Burrell A J M, Coldham I (2010). Curr Org Synth.

[16] Hong A Y, Vanderwal C D (2017). Tetrahedron.

[17] Wang F-X, Du J-Y, Wang H-B, Zhang P-L, Zhang G-B, Yu K-Y, Zhang X-Z, An X-T, Cao Y-X, Fan C-A (2017). J Am Chem Soc.

[18] Hughes J M E, Gleason J L (2018). Tetrahedron.

[19] Kerkovius J K, Kerr M A (2018). J Am Chem Soc.

[20] Wang Y, Hennig A, Küttler T, Hahn C, Jäger A, Metz P (2020). Org Lett.

[21] Yang B, Li G, Wang Q, Zhu J (2023). J Am Chem Soc.

[22] Irie Y, Yokoshima S (2024). J Am Chem Soc.

[23] Burrell A J M, Coldham I, Oram N (2009). Org Lett.

[24] Burrell A J M, Coldham I, Watson L, Oram N, Pilgram C D, Martin N G (2009). J Org Chem.

[25] Alkayar Z T I, Coldham I (2019). Org Biomol Chem.

[26] Saruengkhanphasit R, Collier D, Coldham I (2017). J Org Chem.

[27] Coldham I, Burrell A J M, Guerrand H D S, Oram N (2011). Org Lett.

[28] Hajra S, Maity S, Roy S, Maity R, Samanta S (2019). Eur J Org Chem.

[29] Sharma B M, Yadav M, Gonnade R G, Kumar P (2017). Eur J Org Chem.

[30] Hajra S, Roy S, Maity S (2017). Org Lett.

[31] Coldham I, Jana S, Watson L, Pilgram C D (2008). Tetrahedron Lett.

[32] Furnival R C, Saruengkhanphasit R, Holberry H E, Shewring J R, Guerrand H D S, Adams H, Coldham I (2016). Org Biomol Chem.

[33] Tyler A R, Ragbirsingh R, McMonagle C J, Waddell P G, Heaps S E, Steed J W, Thaw P, Hall M J, Probert M R (2020). Chem.

