# Supplementary material for: Preparation of spirocyclic oxindoles by cyclisation of an oxime to a nitrone and dipolar cycloaddition
Source: Beilstein J Org Chem. 2025 Sep 11;21:1890–6. doi: 10.3762/bjoc.21.146 (PMC12434923; doi:10.3762/bjoc.21.146)

BEILSTEIN JOURNAL OF ORGANIC CHEMISTRY

Supporting Information

for

Preparation of spirocyclic oxindoles by cyclisation of an oxime to a nitrone and dipolar cycloaddition

Beth L. Ritchie, Alexandra Longcake and Iain Coldham

Beilstein J. Org. Chem. **2025,** *21,* 1890–1896. doi:10.3762/bjoc.21.146

ENaCt protocols, X-ray diffraction data for 5a, and NMR spectra for novel compounds

License and Terms: This is a supporting information file under the terms of the Creative Commons Attribution License (https://creativecommons.org/licenses/by/4.0). Please note that the reuse, redistribution and reproduction in particular requires that the author(s) and source are credited and that individual graphics may be subject to special legal provisions.
The license is subject to the *Beilstein Journal of Organic Chemistry* terms and conditions: (https://www.beilstein-journals.org/bjoc/terms)

Table of contents

1. ENaCt protocols S1
2. X-ray diffraction data for compound **5a** S4
3. NMR spectra for novel compounds S7

1. ENaCt protocols

The crystallization of **5a** was carried out using encapsulated nanodroplet crystallization (ENaCt) protocols.[1] A stock solution of **5a** was prepared in methanol (45.2 mg in 1.2 mL), which was evenly dispensed across twelve separate 1.75 mL screw top glass vials and allowed to evaporate. Each vial contained approximately 3.8 mg of compound. Samples of **5a** were then dissolved in a range of 12 different solvents as outlined in Table S1 below:

Table S1 – The preparation of stock solutions for ENaCt experiments. Asterisks denote solutions where the sample was not fully soluble, therefore, supernatant was taken forward to the ENaCt experiments.

Solvent	Volume of solvent added / µL	Approximate concentration/mg mL^{-1}
dimethylsulfoxide (DMSO)	96	39.1
N,*N*-dimethylformamide (DMF)	96	39.1
methanol (MeOH)	96	39.1
N-methyl imidazole (NMI)	48	78.3
toluene (PhMe)	192*	19.6
1,2-dichloroethane (1,2-DCE)	96	39.1
2-methyltetrahydrofuran (2-Me THF)	96	39.1
chlorobenzene (PhCl)	192*	19.6
tetrathiophene-1-oxide (THTP-1-oxide)	96	39.1
acetonitrile (MeCN)	96	39.1
N-methyl-2-pyrrolidone (NMP)	96	39.1
*N*itromethane (NM)	96	39.1

The stock solutions of **5a** (50 nL) were dispensed via an SPT Labtech Mosquito liquid handling robot into 96-well glass plates (SWISSCI LCP Modular, 100 µm spacer) containing either an appropriate crystallization oil (200 nL) or no oil (Figure S1). Plates were sealed with a 175 µm glass cover slip and allowed to stand undisturbed at room temperature in the dark. After 14 days, plates were assessed visually and by cross-polarised light microscopy for crystal growth. Single crystals of **5a** suitable for single crystal X-ray diffraction studies were grown from NMI (50 nL) encased in a droplet of polydimethylsiloxane (PDMSO) oil (200 nL; Plate 1 well G2; Figure S1).

Plate number														
			Volume of Oil (200 nL)											
	**Volume of Solvent (50 nL)**		**1**	**2**	**3**	**4**	**5**	**6**	**7**	**8**	**9**	**10**	**11**	**12**
**1**	Solvent A	**A**	No oil	PDMSO					No oil	Fomblin-Y				
		**B**	No oil	FC-40					No oil	Mineral oil				
**2**	Solvent B	**C**	No oil	PDMSO					No oil	Fomblin-Y				
		**D**	No oil	FC-40					No oil	Mineral oil				
**3**	Solvent C	**E**	No oil	PDMSO					No oil	Fomblin-Y				
		**F**	No oil	FC-40					No oil	Mineral oil				
**4**	Solvent D	**G**	No oil	PDMSO					No oil	Fomblin-Y				
		**H**	No oil	FC-40					No oil	Mineral oil				

Plate 1														
	**Solvent**		**1**	**2**	**3**	**4**	**5**	**6**	**7**	**8**	**9**	**10**	**11**	**12**
**1**	DMSO	**A**	1	1	1	1	1	1	1	1	1	1	1	1
	DMSO	**B**	1	1	1	1	1	1	1	1	1	1	1	1
**2**	DMF	**C**	1	1	1	1	1	1	1	1	1	1	1	3
	DMF	**D**	1	1	1	1	1	1	1	1	1	1	1	1
**3**	MeOH	**E**	1	1	1	1	1	1	1	2	1	2	1	1
	MeOH	**F**	1	2	2	1	2	1	1	2	2	2	2	2
**4**	NMI	**G**	4	4	4	2	3	2	3	2	2	2	1	1
	NMI	**H**	4	4	4	3	2	2	4	2	3	3	3	3

Plate 2														
	**Solvent**		**1**	**2**	**3**	**4**	**5**	**6**	**7**	**8**	**9**	**10**	**11**	**12**
**1**	PhMe	**A**	2	2	2	2	2	2	2	1	1	1	1	1
	PhMe	**B**	2	1	1	1	1	1	2	2	1	2	2	2
**2**	1,2-DCE	**C**	1	1	1	1	1	1	1	1	1	1	1	1
	1,2-DCE	**D**	1	1	1	1	1	1	1	1	1	1	1	1
**3**	2-Me THF	**E**	1	2	2	2	2	2	1	3	3	3	3	3
	2-Me THF	**F**	1	1	1	1	1	1	1	2	2	2	2	2
**4**	PhCl	**G**	1	2	2	2	2	2	1	1	1	3	1	1
	PhCl	**H**	1	1	1	1	1	1	1	2	2	2	2	2

Plate 3														
	**Solvent**		**1**	**2**	**3**	**4**	**5**	**6**	**7**	**8**	**9**	**10**	**11**	**12**
**1**	THTP-1-oxide	**A**	1	1	1	1	1	1	1	1	1	1	1	1
	THTP-1-oxide	**B**	1	1	1	1	1	1	1	1	1	1	1	1
**2**	MeCN	**C**	1	1	1	1	1	1	1	1	1	1	1	1
	MeCN	**D**	1	1	1	1	1	1	1	1	1	1	1	1
**3**	NMP	**E**	1	1	1	1	3	1	1	1	1	1	1	1
	NMP	**F**	1	1	1	1	1	1	1	1	1	1	1	1
**4**	NM	**G**	1	1	1	1	1	1	1	1	1	1	1	1
	NM	**H**	1	1	1	1	1	1	1	1	1	1	1	1

Figure S1 – The plate layout and associated crystallization results for the ENaCt protocol used, detailing the stock solution as well as the classification of the wells after two weeks. Key: 1 = still solvated; 2 = non-crystalline or amorphous material; 3 = microcrystalline material or small single crystals; 4 = large single crystals.

2. X-ray diffraction data for compound **5a**

Single crystals of **5a** were obtained via EnaCt[1] protocols (Plate 1 well G2) from NMI (50 nL) and PDMSO oil (200 nL). A suitable crystal (0.19 × 0.12 × 0.08 mm) was extracted under Fomblin-YR1800 then mounted on a 35 µm MiTeGen loop before being flash cooled to 150 K before collection using an Oxford Cryosystems CryostreamPlus open-flow N_2 cooling device. Data were collected on a Rigaku XtaLAB Synergy diffractometer equipped with a micro-focus sealed Cu Kα X-ray tube radiation source and a HyPix Arc-100 detector. Unit cell measurement, data collection and data reduction were performed using the software CrysAlisPRO. A numerical absorption correction was applied using gaussian integration over a multi-faceted crystal model.

The structure was solved using SHELXT[2] and refined using SHELXL[3] through the Olex2[4] interface (Figure S2). Crystallographic data for **5a** has been deposited with the Cambridge Crystallographic Data Centre (CCDC) with the code CCDC 2393991. Full refinement details can be found within the Crystallographic Information File (CIF) in the field '_refine_special_details'.

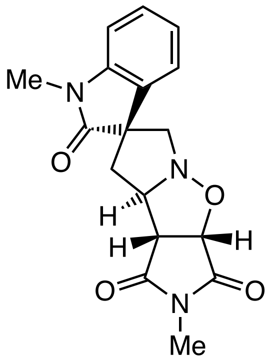

5a

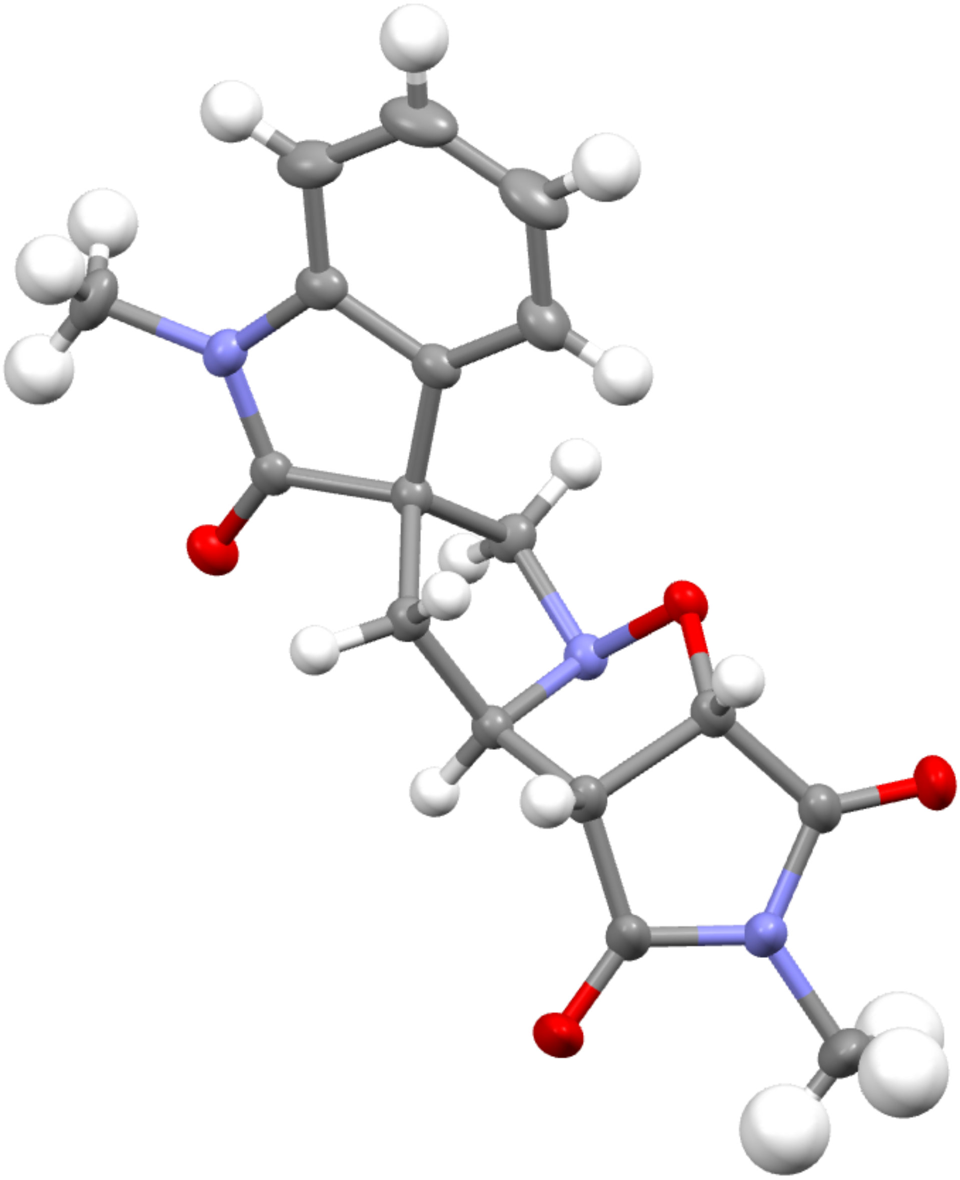

Figure S2 – The crystal structure of **5a** with the anisotropic displacement parameters drawn at 50 %. Key: oxygen – red; nitrogen – blue; carbon – grey; hydrogen – pale green.

Table S2 – Crystal data and structure refinement for **5a**.

Identification code	2023NCS0717
CCDC code	CCDC 2393991
Empirical formula	$C_{17}H_{17}N_3O_4$
Formula weight	327.33
Temperature/K	150.00(10)
Crystal system	monoclinic
Space group	$P2_1/c$
a/Å	8.14960(10)
b/Å	18.27090(10)
c/Å	10.92320(10)
α/°	90
β/°	106.3020(10)
γ/°	90
Volume/Å^3	1561.08(3)
Z	4
ρ_{calc}g/cm^3	1.393
μ/mm^{-1}	0.839
F(000)	688.0
Crystal size/mm^3	0.19 × 0.12 × 0.08
Radiation	Cu Kα (λ = 1.54184)
2Θ range for data collection/°	9.682 to 152.844
Index ranges	-10 ≤ h ≤ 10, -22 ≤ k ≤ 22, -13 ≤ l ≤ 9
Reflections collected	28495
Independent reflections	3166 [R_{int} = 0.0205, R_{sigma} = 0.0097]
Data/restraints/parameters	3166/0/219
Goodness-of-fit on F^2	1.046
Final R indexes [I>=2σ (I)]	R_1 = 0.0341, wR_2 = 0.0891
Final R indexes [all data]	R_1 = 0.0354, wR_2 = 0.0901
Largest diff. peak/hole / e Å^{-3}	0.26/-0.18

References:

1. Tyler A. R., Ragbirsingh R., McMonagle C. J., Waddell P. G., Heaps S. E., Steed J. W., Thaw P., Hall M. J., Probert M. R. (2020), *Chem. 6*, 1755–1765.
2. Sheldrick, G.M. (2015), *Acta Cryst.* A71, 3–8.
3. Sheldrick, G.M. (2008), *Acta Cryst.* A64, 112–122.
4. Dolomanov, O.V., Bourhis, L.J., Gildea, R.J, Howard, J.A.K. & Puschmann, H. (2009), *J. Appl. Cryst. 42*, 339–341.

3. NMR spectra for novel compounds

^{1}H NMR ($CDCl_3$) for compound **5a**

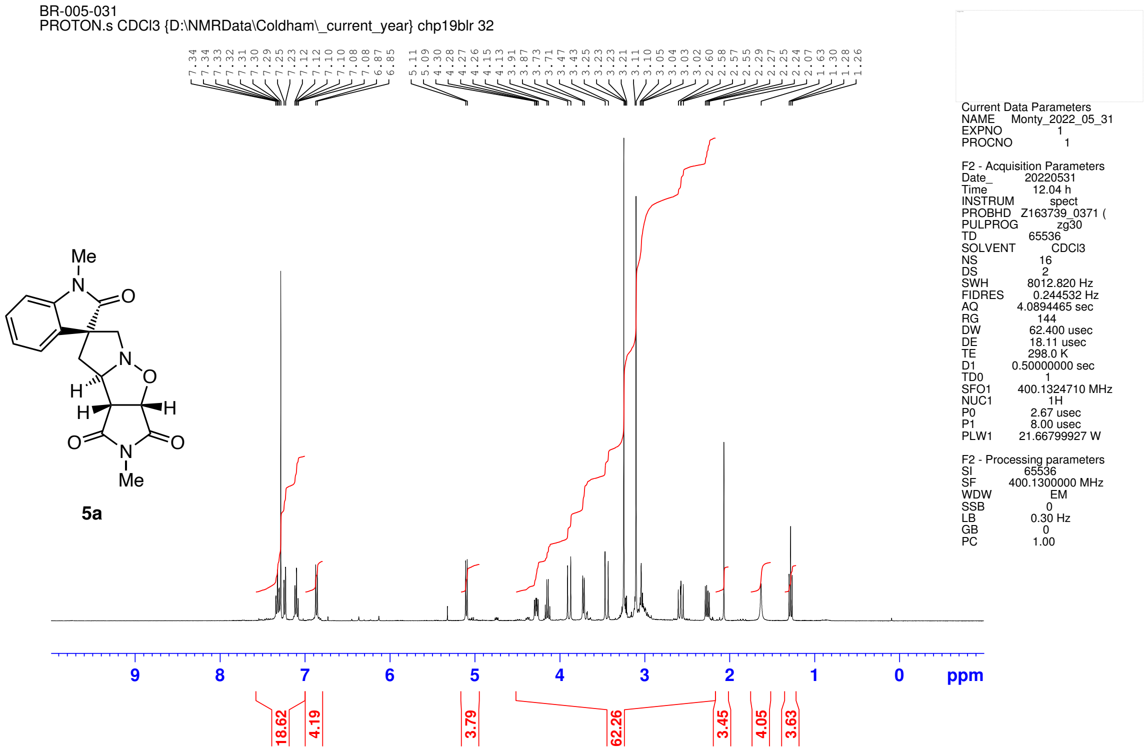

^{13}C NMR ($CDCl_3$) for compound **5a**

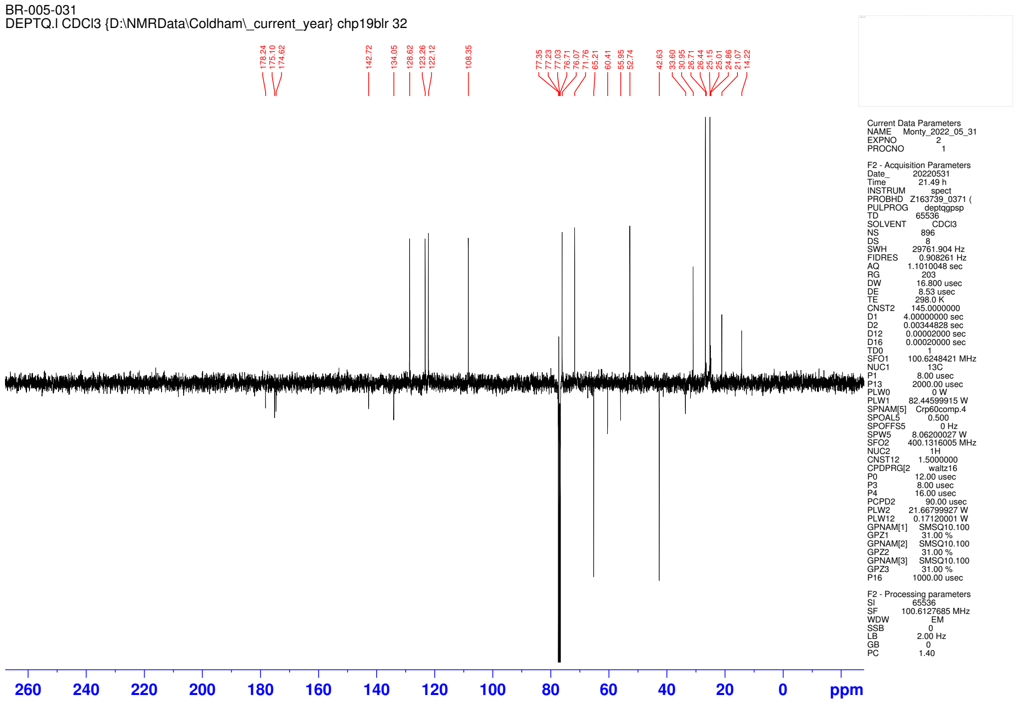

^{1}H NMR ($CDCl_3$) for compound **6**

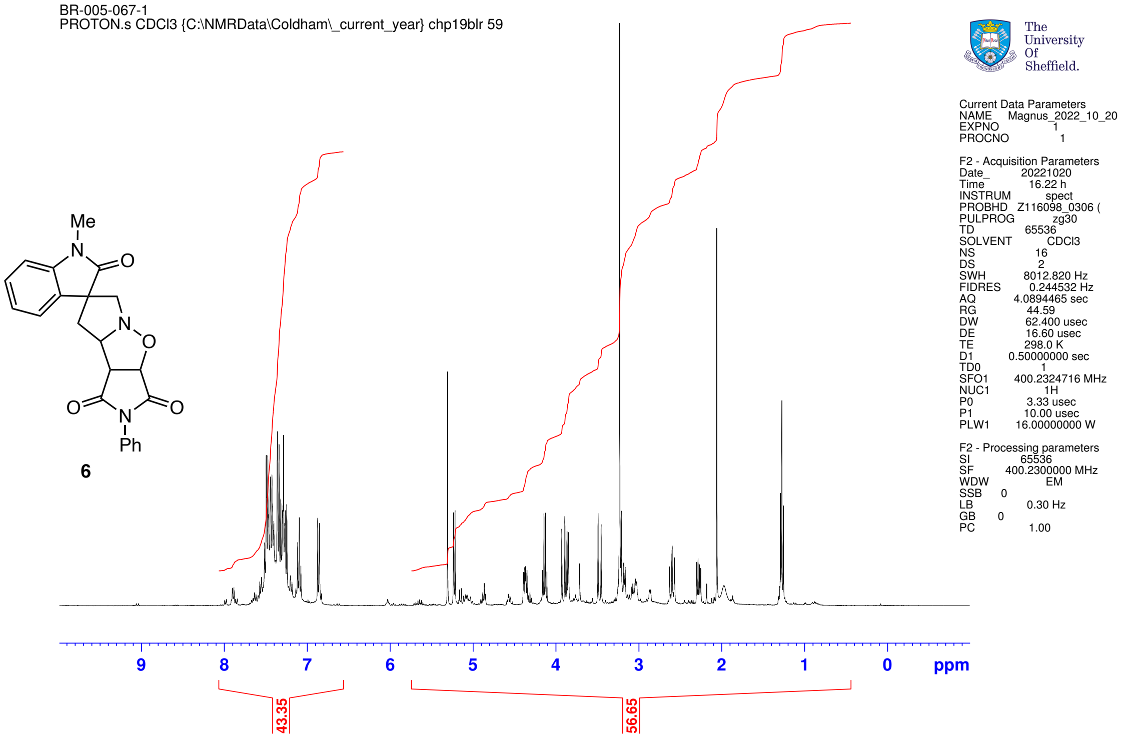

^{13}C NMR ($CDCl_3$) for compound **6**

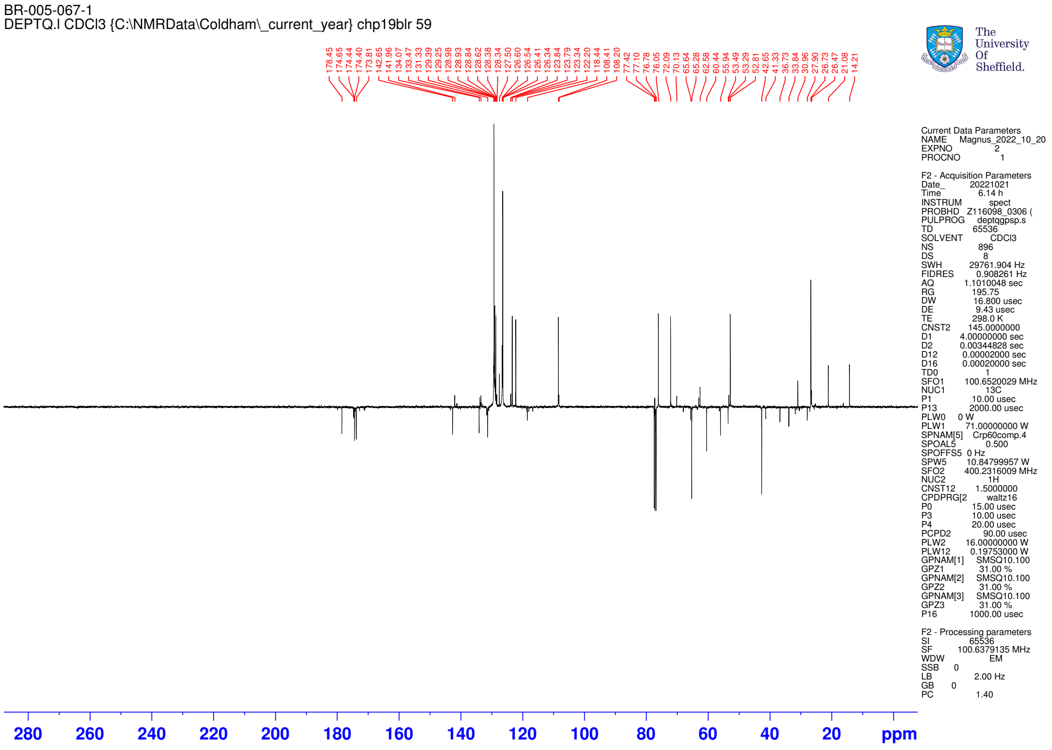

^{1}H NMR ($CDCl_3$) for compound **7**

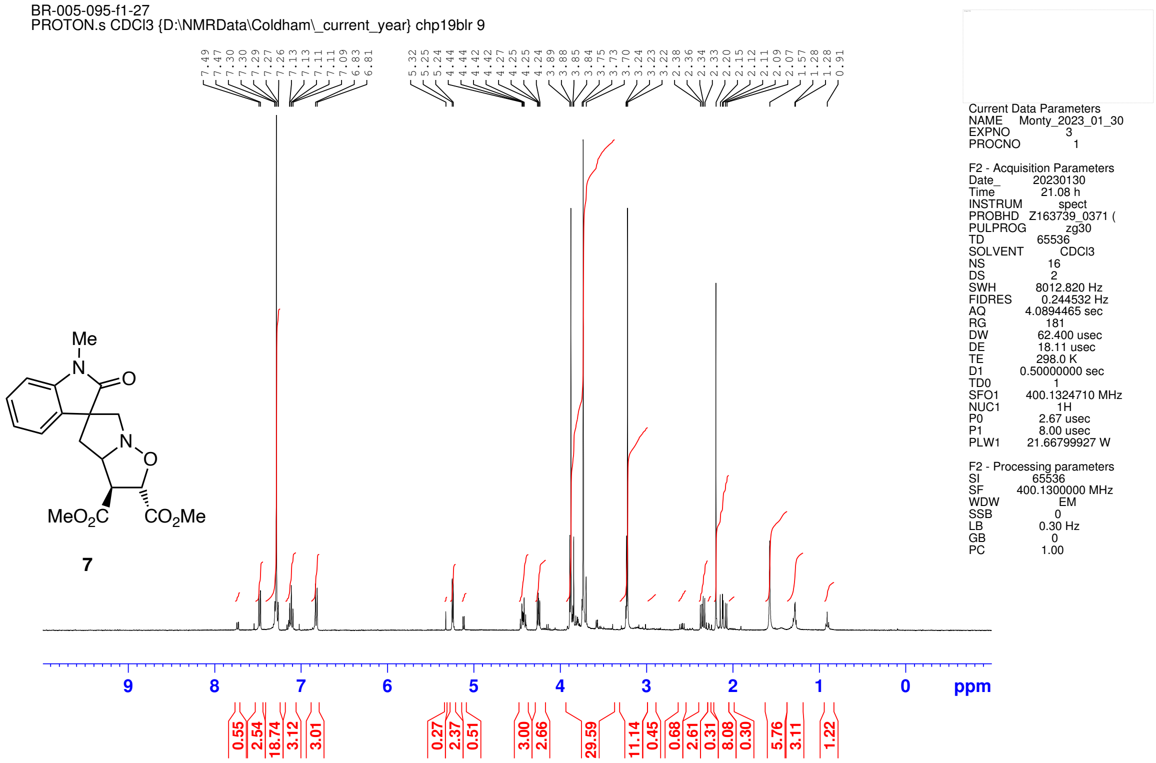

7

^{13}C NMR ($CDCl_3$) for compound **7**

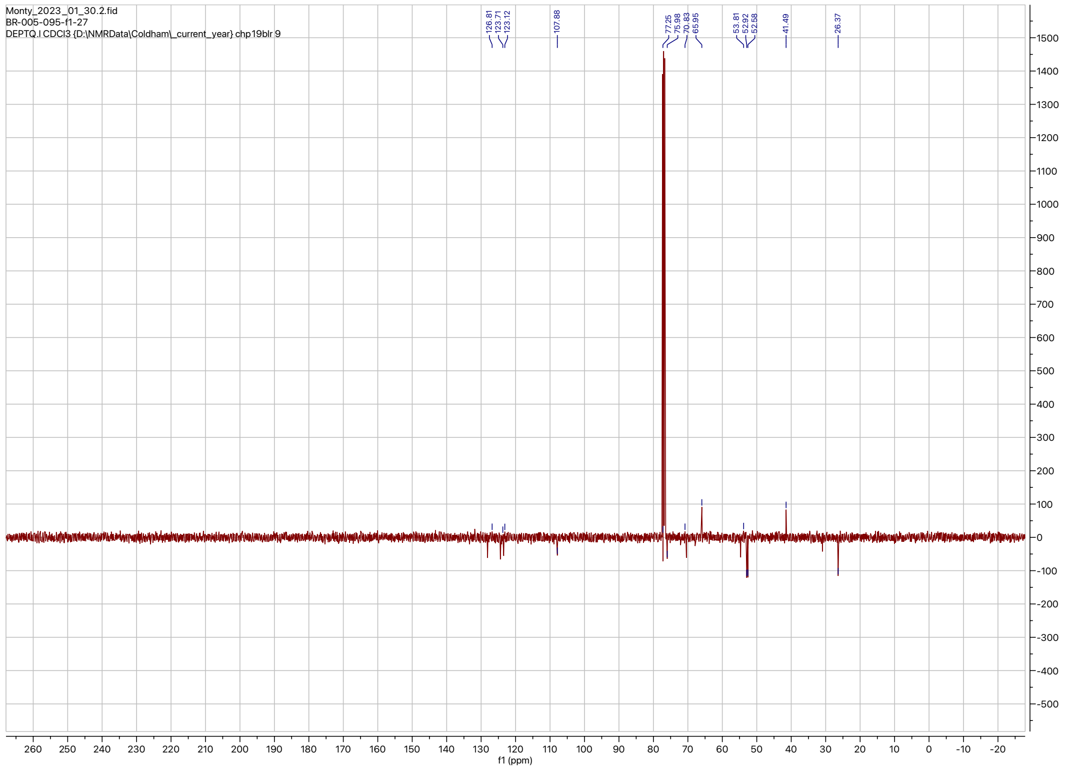

^1H NMR ($CDCl_3$) for compound **8**

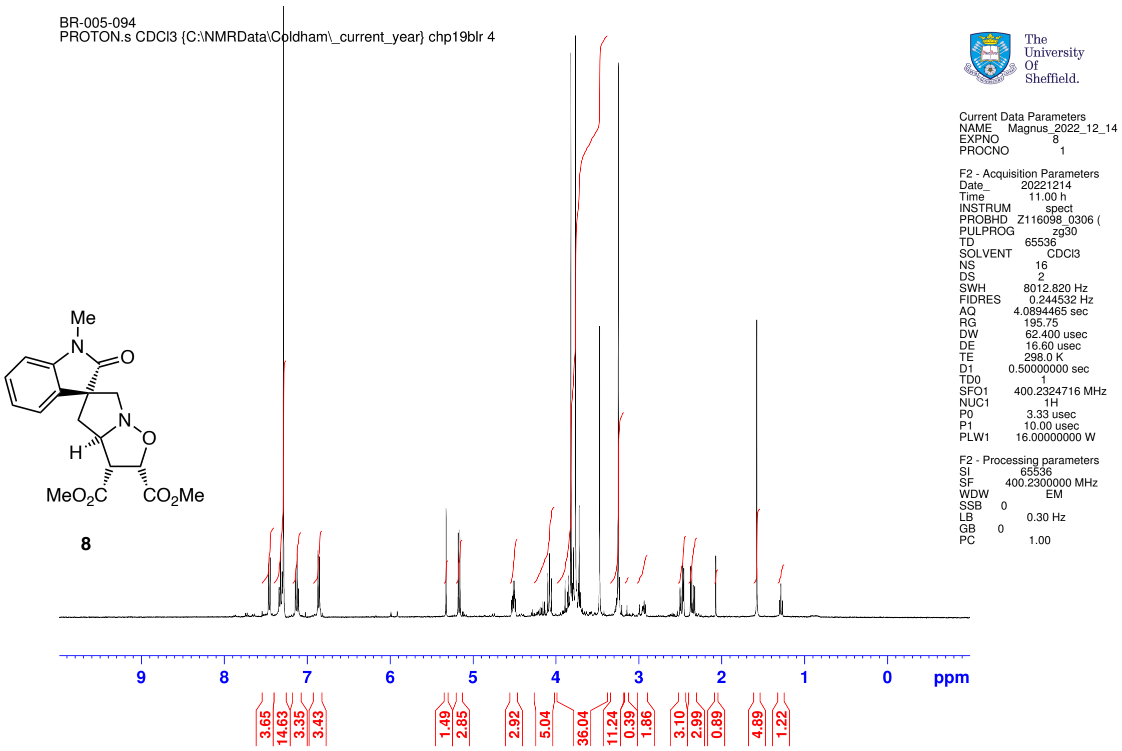

^{13}C NMR ($CDCl_3$) for compound **8**

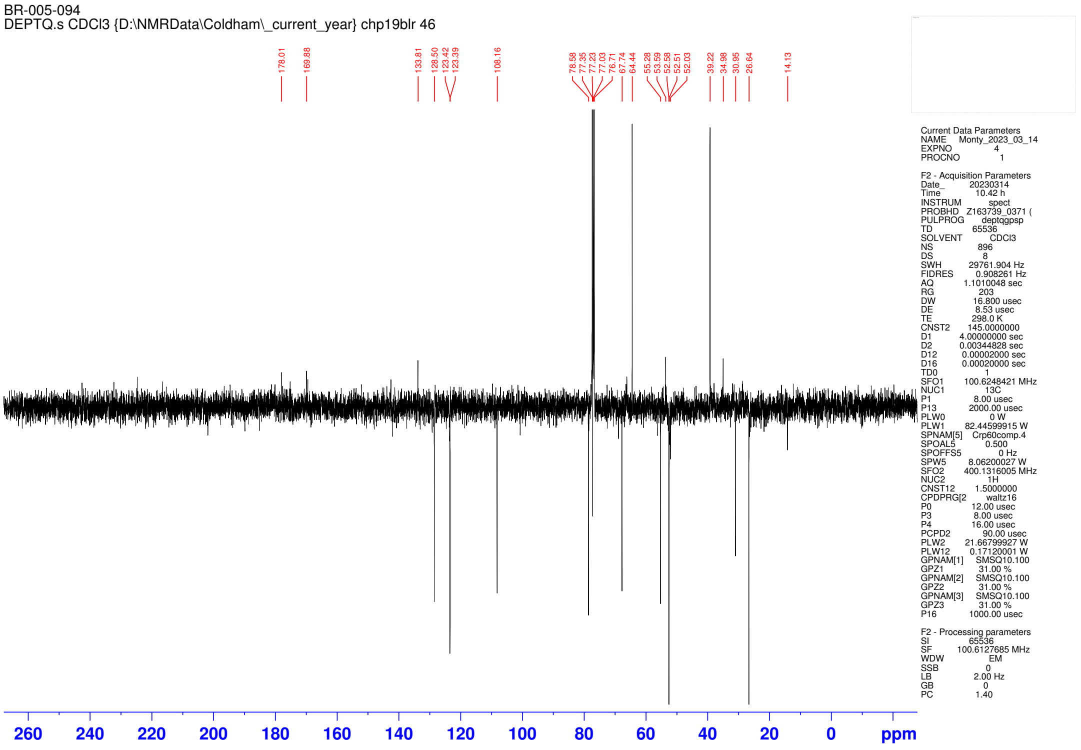

Supplement: File 1 — ENaCt protocols, X-ray diffraction data for 5a, and NMR spectra for novel compounds. [file Beilstein_J_Org_Chem-21-1890-s001.pdf]
